# Current Biological Insights of *Castanea sativa* Mill. to Improve Crop Sustainability to Climate Change

**DOI:** 10.3390/plants14030335

**Published:** 2025-01-23

**Authors:** Tiago Marques, Andrea Ferreira-Pinto, Pedro Fevereiro, Teresa Pinto, José Gomes-Laranjo

**Affiliations:** 1 Centre for the Research and Technology of Agroenvironmental and Biological Sciences, CITAB, Inov4Agro, Universidade de Trás os Montes e Alto Douro, UTAD, Quinta de Prados, 5000-801 Vila Real, Portugal; tpinto@utad.pt (T.P.); jlaranjo@utad.pt (J.G.-L.); 2 Department of Biology and Environment, School of Life Sciences and Environment, University of Trás-os-Montes and Alto Douro, Quinta de Prados, 5000-801 Vila Real, Portugal; al54555@utad.pt; 3 Plant Cell Biotechnology Lab, Institute of Chemical and Biological Technology António Xavier (Green-it Unit), University Nova of Lisbon, 2780-157 Oeiras, Portugal; 4 Department of Plant Biology, Faculty of Sciences, University of Lisbon, Campo Grande, 1749-016 Lisbon, Portugal

**Keywords:** Chestnut (*Castanea sativa*), edaphoclimatic conditions, adaptative plasticity, drought, heat

## Abstract

The sustainability of agriculture is seriously threatened by climate change. In Europe, chestnut ecosystems, which are growing mainly in Mediterranean climate, are facing during summertime increasing of heat and drought stresses. These induce fragilities on trees, leading to a reduction in productivity and predisposing them to pest and disease attacks. The plasticity of chestnut species under contrasting climate is known. Understanding the specific adaptation of cultivars to different climate features is now important to anticipating climate changes. Caucasian Region is considered the origin center of chestnut (*Castanea sativa*), which is characterized by climatic transition from the Mediterranean to the Euro-Siberian area. Mostly, areas of chestnut are concentrated in the countries around the Mediterranean Basin, thriving in regions with humid and Pré-Atlantic bioclimates. In Portugal, more than 95% of the chestnut area is located in the Center and North side of Portugal. This is an anisohydry species, characterized by good hydroplasticity: 90% reduction in A occurs when Ψ_wstem_ drops to −1.25 MPa, and a 50% reduction in A occurs at values of −1.7 MPa. The highest fatty acid contents in chestnut chloroplasts are a-linolenic acid (18:3), ranging between 40 and 50% of the total amount and being the unsaturated/saturated 2.27 for Longal. New strategies are being investigated in order to increase tolerance against those abiotic factors in chestnut species. They include the use of innovative irrigation techniques, which can increase production 22–37%. Fertilization with silicone (Si) has been investigated to promote the tolerance of plants against heat and drought stresses. Breeding programs, mostly (in Europe) against ink disease, have been performed since the middle of the XX century to create new genotypes (such the Portuguese ColUTAD^®^). ClimCast, a network of orchards, was created in Portugal with the aim of responding to the new challenges facing orchards in the context of climate change.

## 1. Introduction

The natural or anthropogenic dispersion of the chestnut genus in various regions had a significant impact on human civilization over the centuries due to its multifunctional relevance, particularly in timber and fruit production [1]. In several regions of Europe, the cultivation of chestnut became dominant and essential for the survival of mountain populations, prompting some authors to refer to it as a “chestnut civilization” [2]. Its occurrence is deeply tied to human activity. In Europe, it is considered a multifunctional species supporting very important chestnut ecosystems (CEs): agro-chestnut ecosystems (ACEs), agroforest-chestnut ecosystems (AFCEs), and forest-chestnut ecosystems (FCEs). ACEs are typically modern orchards with a regular and high-density distribution of relatively young trees (typically less than 50 years old), which is characterized by the continuous agricultural intervention of man. AFCE refers to the irregular and low-density distribution of old trees managed to provide valuable food (such as nuts, edible fungi, honey, and cattle) and wood (firewood, timber) simultaneously. FCEs are forest stands dominated by chestnut trees and are usually managed as coppice forests for timber production, slope protection against natural hazards, wildlife habitat, and recreation [3].

**Figure 1:**
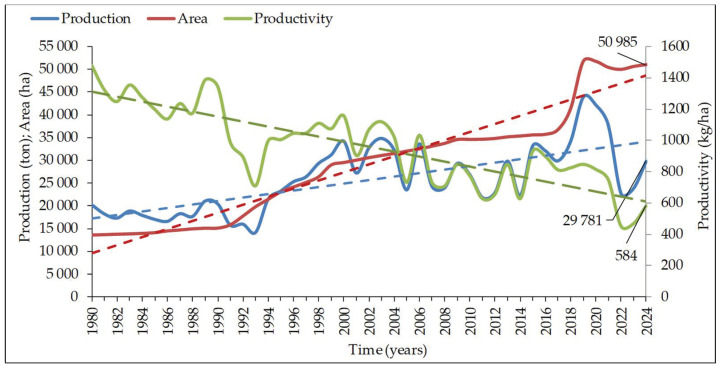
Evolution of area, production and productivity of chestnut in Portugal between 1980 and 2024 [5]. Linear equations: area (red line) = 854.6 year − 2 × 10^−6^, R^2^ = 0.94; production (blue line) = 379.89 year − 734,872, R^2^ = 0.47; and productivity (green line) = −15.983 year + 32,960, R^2^ = 0.70 (green line).

In 2020, chestnut production reached over 2 million t, with a worldwide chestnut tree area of about 600 K ha [4]. According to FAO [4] in Europe, there is an estimated 1.8 M hectare (ha) of FCE, whilst for ACE and AFCE, there is an estimated 0.43 M ha. The area of ACE and AFCE in Portugal is 51 K ha (Figure 1), being the ACE area estimated around 35 K ha. The area increased 3.9 times since 1980, but production only increased 1.9 times, indicating a decrease in productivity and competitiveness [5]. It should be emphasized that the inter-annual variability in the amount of production can be attributed to the climatic conditions of each year, which as we know, are changing.

Fully understanding the impact of climate change on natural ecosystems and society, together with the identification of the dangers and weaknesses involved, constitutes a crucial point for understanding the current climate crisis and for thinking about solutions to mitigate this problem in the sector. According to Perlin at al. [6] if the current temperature trends persist, the 1.5 °C increase, compared with the Industrial Revolution, would be reached before the expected between 2030 and 2050. This escalation is poised to unleash disastrous consequences on both the environment and human society [6]. Climate change generated crop changes, such as an increase in disease mortality, the appearance of new pests, and increasing greenhouse gas (GHG) emissions, necessitating quick farming management decisions [7,8]. For many crops, heat stress and drought stress usually combine [9,10], and the combination of these two types of stress results in greater yield decreases than either stress alone [11]. In addition to decreasing nutrient uptake and photosynthetic efficiency, drought and heat stress cause several physiological and biochemical reactions in plants [12]. The numerous genes involved in stress reactions make it challenging to produce crops that can withstand stress [13].

Mitigation and adaptation methods play a crucial role in combating the effects of climate change [14]. Adaptation measures increase adaptive capacity and reduce vulnerability to climate change effects while also benefiting from favorable climate change possibilities. Future socio-economic scenarios must be considered while developing adaptation strategies to provide professionals with a framework for making adaptation decisions [15,16].

Producers, industry partners, and decision makers aiming to adapt to climate change over multiple seasons should prioritize the consideration of long-term adaptation solutions [17]. While implementing some long-term adaptation strategies may be important, doing so could come at a large socioeconomic cost [18]. The potential of the various adaptation strategies may still be able to prevent more drastic changes in the capability of a given place to produce even when changes in bioclimatic conditions are anticipated to occur in the future [19].

The aim of this work is to provide an overview of the key mechanisms that contribute to chestnut’s tolerance to the combination of heat and drought. Additionally, it will explore management practices that can enhance chestnut resilience and increase productivity in the face of these climatic stresses.

## 2. Knowing *C. sativa* Plasticity Anticipating Climate Changes

### 2.1. Botanical Level

The genus *Castanea*, along with the genus *Quercus* and *Fagus*, is a significant component of the family Fagaceae. *Castanea* is a genus that has shown remarkable adaptability to climatic changes over millions of years, starting from the Tertiary period. The origin region of the genus *Castanea* is hypothesized to be in the Eastern area of Asia from where a migration of species via Europe to North America happened during the Eocene [20,21,22]. According to these authors, the initial diversification of *Castanea* in eastern Asia during the Eocene period led to the spread and divergence of species between Asia and Europe/North America, with further differentiation among European and North American species in later Eocene times [23]. The genus is divided into three sections: Eucastanon, Balanocastananon, and Hypocastanon, with the Eucastanon section containing the most economically valuable and representative species [24].

The Eucastanon section includes the species that are characterized by three nuts per burr [25]. *Castanea* species can be found in Asia, Japan, North America, and Europe [26]. The European species is *Castanea sativa* Mill., which is nowadays unique in Europe, and resisted to the last Würms glaciation [27].

Studies carried out by Aravanopoulos, Mattioni et al., and Pereira-Lorenzo et al. [28,29,30] revealed a significant level of genetic variability within the species, highlighting the existence of the Greece and the Iberian Peninsula clusters. Later, Mattioni et al. [31], through a study based on around 1600 tree samples from 73 locations in 14 European countries (located from East to West), concluded that there are three large clusters of genetic proximity: (1) Western Europe, (2) the southern part of Central Europe, and (3) Eastern Europe. In turn, in the Iberian Peninsula, two major centers of origin of the genetic variability of chestnut varieties were found, one in the northwest region and the other in the central region [30], both consisting of genotypes identified with Longal cultivar. Later, due to the colonization, which occurred from these two centers to the current chestnut areas, genetic differentiation increased as a result of the combined action of factors such as climate and soil, mutations, spontaneous hybridizations, and the propagation processes used [31,32].

The wide distribution of this species across Southern Europe and Minor Asia was associated with its genetic variability [33]. This variability, especially found in the Caucasian region, was consistent with ecological, physiological, and morphological diversity. This region, which is considered the center of *Castanea sativa* origin, is characterized by climatic transition from the Mediterranean to the Euro-Siberian area [33,34]. Two distinct population types were identified: (1) an eastern population adapted to wet conditions with higher photosynthetic rates and transpiration, along with greater carbon isotope discrimination (D), compared to (2) a Mediterranean population adapted to drought. This highlights how the common adaptive mechanisms, both genetic and physiological, of *Castanea sativa* enable it to colonize a wide range of site conditions in Europe [33].

### 2.2. Ecological Level

**Figure 2:**
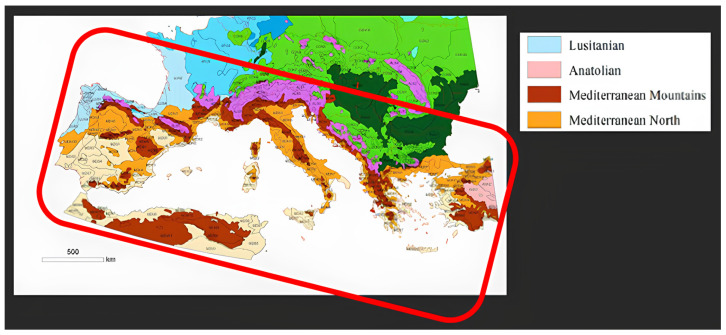
Environmental stratification of the Mediterranean countries. Inside the red box are the regions with adequate environmental zones for chestnuts grown for fruit production. Adapted from [36].

*C. sativa* is widely distributed in the deciduous forests of the temperate regions of the Northern Hemisphere [35], between 30° and 50° N (Figure 2), surrounding the Mediterranean Basin. It thrives in regions with humid and Pré-Atlantic bioclimates, with an Emberger coefficient of 120 to 180, corresponding to the environmental zones of Anatolian, the Lusitanian Mediterranean Mountains, and the Mediterranean North [36], suggesting that this species has considerable plasticity, which is advantageous for breeding programs aimed at improving adaptation in the context of climate change.

The climate of Portugal is temperate with Mediterranean features, which is in line with the requirements of chestnut cultivation [37,38]. More than 90% of chestnut trees are cultivated in the Portuguese territory north of the Tagus River [39]. In the North Hemisphere, the period of the vegetative cycle mostly takes place, between May and October, which corresponds mostly to the summertime. Normally, only 17% of the year’s total rainfall (923 mm), almost 67% of the total degree days (3230 °D), and 92% of total xerotermic index (XI) (97) occur during this period [40]. XI represents the level of the environment’s aridity in a region, that is the sum of days in which total rainfall is lower than the double of the mean temperature over the given period [41]. This indicates that the vegetative cycle of chestnut is deeply influenced by hot and droughty summertime [42].

**Figure 3:**
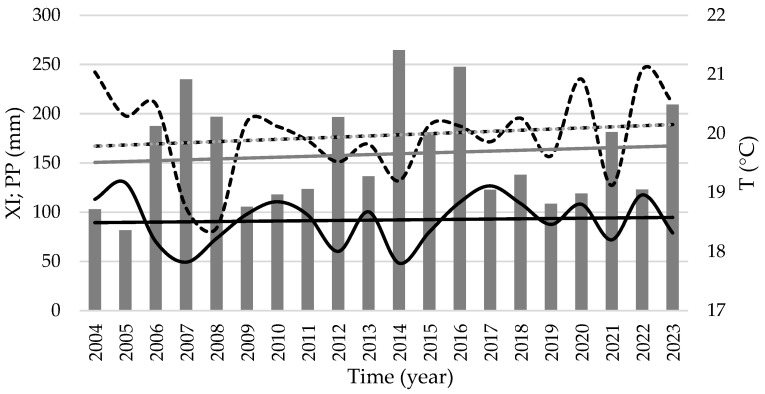
Mean temperature (T, dashed line), total precipitation (PP, bars), and xerothermic index [black line; XI = Σ(2Tmean − PPmean); if 2Tmean ≤ P, consider 0)] occurred during summer season (1st May till 30th September) every year between 2004 and 2023, in Center and North of Portugal [43]. Linear equations: temperature = 0.0193 year + 19.77, R^2^ = 0.03; precipitation = 0.885 year + 149.74, R^2^ = 0.01, and XI = 0.279 year + 89.15, R^2^ = 0.005. Adapted from [44].

In this chestnut growth season, between 2004 and 2023, mean air temperature (T) changed from a minimum of 18.4 °C (2008) to a maximum of 21.0 °C (2004), with a trend of 0.02 °C per year (Figure 3). The rainfall changed between a minimum of 81.8 mm (2005) and 265 mm (2014), with a trend of 0.89 mm/year. The xerothermic index changed between 48.3 units (2014) and 130 units (2005), increasing 0.27 units per year, highlighting the increase in aridity of the season.

### 2.3. Morphological and Phenological Level

Climate change may represent a threat to chestnut growth. The reason is that temperature plays a crucial role in various processes, namely at the level of morphology, physiology, biochemistry, and genetics [37].

Because leaves are the most flexible organs responding to environmental conditions [45], their structure reflects more clearly (than the stem and roots) the severity effects of summer heat and drought [46]. Leaf development is not only influenced by radiation, but also significantly affected by heat accumulation [1]. This can lead to either the anticipation or delay of various phenological stages (see below). This, in turn, can have significant consequences on productivity and fruit characteristics [47].

**Figure 4:**
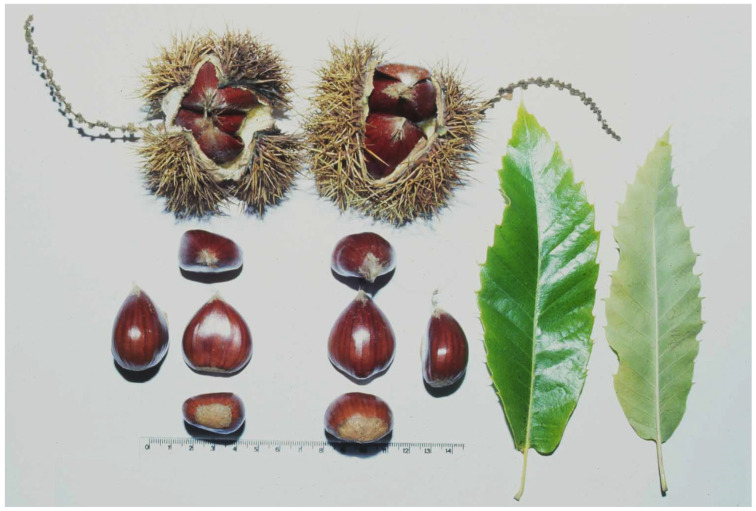
Leaves, burr, catkins and nuts from Longal cultivar, the ancient cultivar in Iberian Peninsula and one of the most popular in Portugal (the scale is presented in cm) [48].

The chestnut leaves feature approximately 20 pairs of secondary veins that alternate in a parallel arrangement and exhibit undulations in the spaces between the main and secondary veins (Figure 4). The veins are particularly noticeable in their abaxial side. These leaves are deciduous, petiolate, and oblong-lanceolate in shape, with an acute or acuminate vertex and a serrated margin. According to [48], in a study of 33 Portuguese chestnut cultivars (*C. sativa*) to analyze leaf morphology, the mean length was 154.8 mm (ranging from 127.8 mm to 177.7 mm) and the width averaged 55.1 mm (ranging from 47.0 mm to 69.8 mm). Regarding the number of secondary veins, chestnut leaves typically have an average of 17.1, with variations ranging between 14.9 and 19.3.

**Figure 5:**
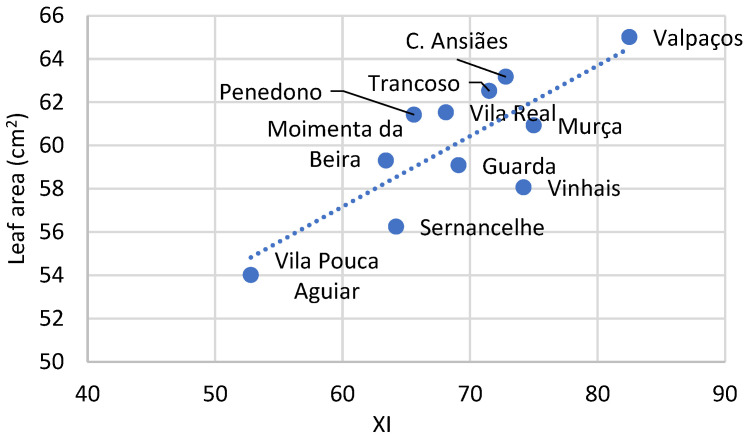
Influence of xerothermic index [XI = Σ(2T_mean_ − PP; if 2T_mean_ ≤ P, XI = 0)] on leaf area (LA) of adult chestnuts from the Longal cultivar. Localities in the figure correspond to a Portuguese regions where samples were collected (tendency line is characterized by linear equation LA = 0.3261XI − 37.611, R^2^ = 0.63). Adapted from [48].

Since leaves play a crucial role in the plant’s life cycle, and since the vegetative cycle is deeply influenced by the xerothermic index (Figure 5), their form must be optimized to facilitate processes such as photosynthesis, transpiration, and nutrient absorption, among others, to support plant growth and development [49]. The ability of different plant species to withstand varying levels of stress, such as heat and drought, is directly linked to their unique leaf forms and physiological adaptations [50]. Previous studies identified significant correlations between plant cultivar, resilience to cold and drought, rootstock strength, and the density of stomata in the leaves [51,52,53], as it is demonstrating for the Longal cultivar.

Gomes-Laranjo et al. [48] studied leaf thickness in Portuguese cultivars, ranging from 152.2 µm (Amarelal cultivar) to 264.6 µm (Martaínha cultivar). The thickness of the Longal cultivar was 196.4 µm. Palisade parenchyma tissue, formed by compact cells located immediately below the epidermis of the leaf’s upper side, is mainly responsible for the leaf’s capacity for photosynthetic activity. This indicates that plants with less thick parenchyma are more adapted to shade than sun. It may also indicate that these plants will find it more difficult to adapt to warmer environments (greater light intensity) than plants with thicker parenchyma. Regarding this tissue, the cultivar Judia had the thickest palisade mesophyll with 129.6 µm, followed by Martaínha, whose measurement was 121 µm, while in the Longal cultivar this tissue was the thinnest with only 89.2 µm. The ratio between palisade and spongy mesophyll changed between 1.31 (Longal cv.) and 1.98 (Judia cv.) [48]. Despite having thicker palisade parenchyma, Judia’s ability to adapt to intense light is not just affected by this. According to field research, Longal does better in high-light and stressful areas (Parada), whereas Judia does better in more moderate conditions. This is most likely caused by variations in the antioxidant metabolites, enzyme performance, and membrane composition [48] that enable Longal to better withstand photo-oxidative stress in Parada. Because of this, parenchyma thickness is just a partial measure of adaptation; other physiological and biochemical variables are more crucial for field performance.

Trichomes are epidermal cells transformed into small hairs that can be unicellular or multi-cellular [54]. Leaf trichomes act as an effective barrier against solar radiation. They increase light reflectivity to reduce photoinhibition, cool leaves to maintain photosynthetic temperatures, and protect from insect attacks [55,56] and higher temperatures due to climate changes. High trichome densities may improve drought resistance by thickening the leaf boundary layers and reducing transpiration [56]. According to observations of several types of Portuguese chestnut trees, the Martaínha cultivar has non-glandular trichomes on the epidermis of the abaxial page of the leaf mesophyll [48]. Concerning stomata density, values ranged between 298 stoma/mm^2^ (Aveleira cv.) and 468 stoma/mm^2^ (Trigueira cv.).

Ciordia et al. [57] concluded that a limited water supply reduces plant water potential, increases root development, decreases leaf area, and changes leaf morphology [58]. Temperate-climate trees, such as chestnuts, need winter cold to meet their chilling requirements during winter dormancy, which influences the budburst [57]. During warmer spring temperatures, plants with accumulated exposure to low temperatures can appropriately set inflorescence production [59]. The chestnut tree has been linked to a moderate chilling accumulation, which might be less than 90 chilling portions (or dynamic model), which is a model that compensates for the fluctuations of the temperature, i.e., it calculates chilling accumulation (chill portions) using a range of temperatures [60]. Following that, heat accumulation acts as a phenology booster, regulating the release from endo-dormancy after the accumulation of sufficient cold units during the winter as well as the release from eco-dormancy, whose duration is dependent on forcing units accumulated from endo-dormancy to the flowering stage [59]. Budburst timing, for example, is influenced by exposure to cold temperatures (chilling) to break dormancy, followed by ideal temperatures to promote plant growth in the spring [61]. Different types of chestnut may have different phenological timings under the same edaphoclimatic conditions, which are impacted by genetic traits [62].

Extremely high summer temperatures may limit the yield potential of chestnut trees. Warmer temperatures signal the onset of vegetative activity, resulting in the progression of phenological stages and a rise in disease and pest incidence [63]. When the air temperature rises above 32 °C, as it does throughout the summer [42], this species may show thermoinhibition. In Portugal, the decay of about 50% in the harvest of 2022, due to the high percentage of aborted nuts, was attributed to the heat wave in July, which affected pollination (many days with maximal temperatures around 40 °C in July) temperatures, even if only for a few days, which is excessive, resulting in stress and consequently low crop yields [64], despite that in the period May to September, the mean air temperature was 21.1 °C and total precipitation was 123 mm (see Figure 1 and Figure 3). On the other hand, in 2019, Portugal had the largest production ever obtained, which was associated with the more favorable temperature between May and September (19.6 °C) and 109 mm of the total precipitation in the same period.

**Figure 6:**
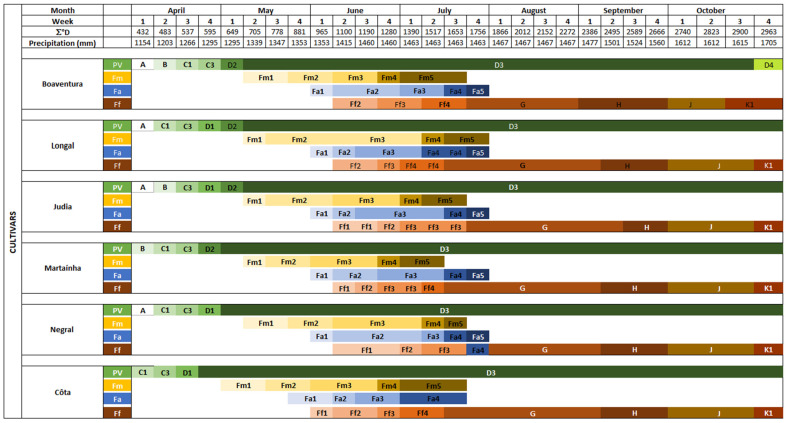
Phenological clock of the Portuguese cultivars (Boaventura, Longal, Judia, Martaínha, Negral, and Côta) as was observed in 2018. Vegetative growth (letters A, B, C, and D), male catkins (letters Fm); androgynous catkins (letters Fa); female growth (letters Ff, G, H, J, and K); adapted from [65].

In Portugal, bud break occurs normally during the second fortnight of April, corresponding to around 200 °D. Depending on the cultivar, the cycle lasts 23 to 26 weeks from 2700 °D to 2900 °D [65] (Figure 6). Full female bloom occurs at 800 to 1000 °D of accumulated heat, and nut growth starts between 2000 °D (Amarelal cultivar) and 2400 °D (Judia cultivar). Some studies have shown that variations in °D due to climate change can lead to the desynchronization of flowering times and, consequently, production [63].

### 2.4. Ecophysiological Level

Concerning main Portuguese chestnut varieties, the overall highest photosynthetic rates (*A*_100_) were found with air temperatures around 25 °C (T_100_) [66]. T_100_ ranged between 22 °C (Lada cv.) and 29 °C (Boaventura), being for Longal 23 °C, while for Judia it was 25 °C. Concerning heat tolerance, the photosynthetic rate decayed 50% when temperatures were around 37 °C (T_M50_). Specifically, for Longal, T_M50_ was around 40 °C, while for Judia it was 36 °C. *A*_100_ is normally obtained in September (25.4 °C), being 15% higher than those measured in July (29.4 °C). In Portugal, September corresponds to the month when nuts start to grow inside the small herbaceous burs, while July is the month of pollination.

A wide range of precipitation values between 600 and 1600 mm is recognized as evidence of chestnut plasticity [67]. On the other hand, the length of the drought period is pointed out as one of the key climatic constraints for chestnut growth, as it can be significantly disadvantaged when drought occurs for more than two months in a row, which is particularly typical in Mediterranean climates. Reduced precipitation causes soil water deficits and plant water stress, impacting plant growth, development, and vigor. This results in smaller organs, limits the size and quantity of individual leaves, inhibits flowering, and affects fruiting formation [59,68,69]. These effects are reflected in plant productivity and fruit qualities and increasing the susceptibility of trees to disease [27].

Chestnut is an anisohydry species, i.e., this type of species has more variable leaf water potential and keeps their transpiration and photosynthesis high even when they present progressively lower water potential (Ψw). When faced with a water deficit, they lower their stem water potential (Ψw_stem_), but continue to transpire to maintain photosynthesis, waiting for the water conditions to improve. When faced with a drought, these plants consume much more water than isohydric plants. If the soil becomes too dry, the plants become water-stressed and die.

Water potential is mainly the result of two components: the osmotic potential (Ψs) and the pressure potential (Ψp) [70]. The stem water potential that provides the maximum rates of photosynthesis (A) (9–11 µmol CO_2_·m^−2^·s^−1^) is around −1.2 and −0.5 MPa [71]. It is also worth noting the hydroplasticity demonstrated by this species, as a 90% reduction in A, occurs when Yw_stem_ drops to −1.25 MPa, and a 50% reduction in A occurs at values of −1.7 MPa [71]. So, these authors suggest that the range of Ψ_Wstem_ (−0.94 to −1.15 MPa) should be considered the reference range for water management in this species. For this range of Ψ*_W_*, a value of Ψs ≈ −1.4 MPa was found, corresponding to a value of Ψp ≈ 0.4 MPa [71]. According to the findings of Tiago et al., the Judia cultivar exhibited values of 0.6 MPa and photosynthetic rates of 14.5 µmol CO_2_·m^−2^·s^−1^ in areas that had suitable water conditions. In contrast, it had water potential values of 1.7 MPa and a reduction of almost 42% (8.5 µmol CO_2_·m^−2^·s^−1^) in areas with less favorable conditions [72].

**Figure 7:**
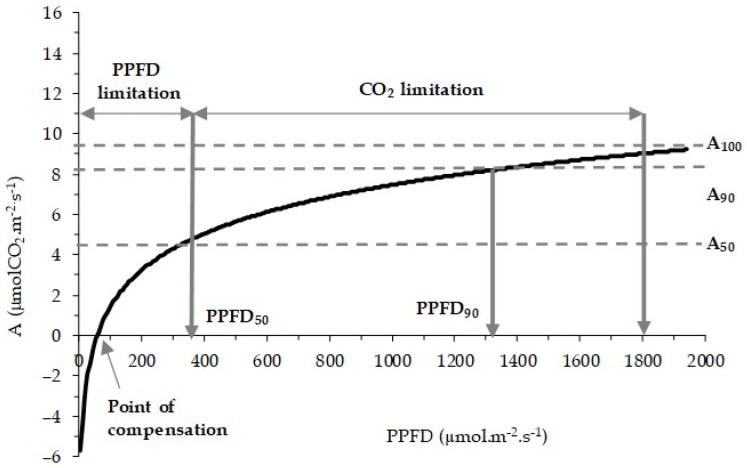
Influence of radiation (PPFD) on the rate of net photosynthesis (A) in the main Portuguese chestnut varieties. Trend line characterized by the logarithmic equation: A = 2.6338ln(PPFD) − 10.725, R^2^ = 0.52. Adapted from [71].

*C. sativa* is a species with half-light characteristics [71], reaching 50% of the maximum rate of net photosynthesis at radiations of about 325 µmol·m^−2^·s^−1^ (PPFD50), suggesting that this is the range (0 to 325 µmol·m^−2^·s^−1^) which corresponds to the limitations of the photosynthetic apparatus due to radiation (Figure 7). The compensation point is about 60 µmol·m^−2^·s^−1^, with a respiratory rate calculated at 10.8µ molCO_2_·m^−2^·s^−1^. Species from temperate climates, such as Portugal, are typically adapted to lower light levels and moderate temperatures due to their evolutionary history. This species may experience photosynthetic stress when exposed to high light intensities that are outside of its range of adaption. This stress can be caused by processes including photosynthesis saturation and an overabundance of free radicals, which damage cells. Plants more suited to a temperate environment may lack the mechanisms necessary to control excessive light in warmer, brighter regions. This leads to photo-oxidative damage, which lowers the plants’ ability to perform photosynthesis and, ultimately, their productivity.

According to previous research conducted with Portuguese cultivars, 90% of the maximum *A* (around 9.5 µmol·CO_2_·m^−2^·s^−1^) is achieved at 1300 PPFD, which is almost half the total sunlight intensity [71]. Half of *A* occurs under 300 PPFD, indicating too that limitation of radiation on the photosynthetic apparatus only appears for radiation below that, being the compensation point around 60 PPFD [50].

### 2.5. Biochemical Level

Lipids play an important role in maintaining an adequate fluid level in the membranes, which is a consequence of the unsaturation level of their fatty acids [73,74,75]. The state of the lipids plays an important function in the temperature response at the thylakoid membranes since it might function as a buffer in a determined range of temperatures.

According to a study conducted with chestnut chloroplasts isolated from leaves of Aveleira, Longal, and Judia harvested in trees from the same orchard and at the same time, the highest fatty acid contents in chestnut chloroplasts are α-linolenic acid (18:3), Aveleira cv. having 41%, Longal cv. 42%, and Judia cv. 47%, and palmitic acid (16:0), Aveleira cv. having 30%, Longal cv. 24%, and Judia cv. 26%. The unsaturated/saturated fatty acid ratio was the lowest in Aveleira cv. at 1.86, 2.27 for Longal cv., and 2.40 for Judia cv. [76]. The adjustment in unsaturation degree by changing the membrane composition to maintain the membrane fluidity is of great importance since it affects the membrane proteins activity and functionality much before than membrane integrity and then the adaptability to changing temperature [76].

Lauteri et al. [33], in a study carried out with young wild populations of chestnut (*C. sativa*) from six climate-contrasting regions of Europe (Málaga, Coruña, Sicily, Piemonte, Central Macedonia, and Northern Macedonia), a xerothermic index (XI) ranging between 0 (Piedmont, Italy) and 118.2 (Málaga, Spain), and combining the effect of temperature (25 and 32 °C) with a water regime (full irrigation and dry regime), concluded that these genotypes presented significant variability concerning to carbon isotopic discrimination (Δ^13^C), and therefore, consequently, variation in adaptive capacity to drought [33]. Concerning the combination of 32 °C with a dry regime, the Δ^13^C mean values of the six populations varied between 23.2% (Northern Macedonia, T25D) and 25.1% (Central Macedonia, T25W). They concluded that populations originating from dry sites (Málaga, Sicily, and Central Macedonia) generally showed higher values and phenotypic plasticity of Δ^13^C than populations from wet sites. So, *C. sativa*’s adaptation mechanisms to drought involve genetic and physiological determinants, allowing it to colonize a wider range of locations. This value is positively correlated with intracellular (Pi) and air (Pa) CO_2_, which, by its way, is correlated with the stomatal conductance and the water use efficiency [77]. When the ratio of Pi/Pa is 1, the value of Δ^13^C approaches 27%, while it drops to 4.44 when the Pi/Pa ratio is 0. For instance, when plants are under drought conditions, which induce stomatal closure, Δ^13^C will increase. Data collected from 14 chestnut cultivars in Trás-os-Montes between pollination and nut maturation (from early July to the first fortnight of October) show that Pi/Pa ranged from 0.70 (at an optimal air temperature of 25 °C) to 0.64 (at a high air temperature of 37 °C) [78].

Gomes-Laranjo et al. [66] reported that the mean photosynthetic pigment content in Portuguese varieties is approximately 110.7 µg·cm^−2^, with the highest value observed in Judia at 142.6 µg.cm^−2^ (Table 1). The mean Chla/b ratio in chestnut, an important parameter to characterize shade and sun leaves, was calculated to be 4.2, with the lowest ratio observed in leaves from Judia [66,79]. According to these findings, the Judia cultivar, which has a lower ratio of chlorophyll a/b, has shaded leaves, or leaves that are better suited to less intense light. This supports the previous theory that that this cultivar will struggle more than Longal and Martaínha in warmer climates and with higher light intensity.

The plant nutrient uptake is the result of the absorption, translocation, and incorporation into the plant’s organs and its efficiency can be correlated with the dry matter production. Soil’s moisture influences the absorption of nutrients and consequently positively affects the plant’s nutrition and fruit quality (Table 2) [46].

Environmental factors influence the reproductive and physiological cycles of chestnut trees, as well as their fruit yields and quality characteristics. According to Martínez et al. [80], antioxidant capacity was negatively affected by maximum temperature and showed significant correlations with altitude, rainfall, and the average period of sunlight. The study indicated that antioxidant activity seemed to increase in the coldest conditions. Furthermore, it has been observed that geographic and climatic factors affect antioxidant capacity, not limited to chestnut cultivars [80].

A study observed that the concentration of phenolic compounds, with their antioxidant potential, was influenced by some factors such as cultivar and climatic conditions. The analyses were conducted using samples collected from different ecotypes of *C. sativa* cv. Judia in the Trás-os-Montes region. The antioxidant capacity was evaluated using several biochemical assays: 2,20-azinobis-(3-ethylbenzothiazoline-6-sulphonic acid) (ABTS) and 2,2-diphenyl-1-picrylhydrazyl (DPPH) radical scavenging activity, ferric reducing antioxidant power (FRAP), and inhibition of oxidative hemolysis in erythrocytes, to evaluate the protective effect of the extracts on hemolysis by peroxyl radical scavenging activity. Total phenolic content varied from 9.6 mg/g of gallic acid equivalent in the hottest ecotype, Murça, to 19.4 mg/g in the coldest ecotype, Valpaços. Martínez et al. [80] highlighted that chestnuts harvested in colder climates exhibited higher values of total polyphenolic content and antioxidant capacity compared to those from warmer environments.

Additionally, several studies showed strong associations between total flavonoid concentration and altitude, precipitation, and the average period of sunlight. The overall flavonoid concentration, however, exhibited a negative correlation with the maximum temperature [80]. Zoratti et al. [81], highlighted that light is one of the most significant environmental factors influencing the formation of flavonoids in plants, indicating that chestnuts produce more flavonoids in the presence of increased sunlight and rain.

The high antioxidant capacities of some chestnut species may be influenced by the increased UV light in places with higher altitudes. It is known that a number of variables, including climate, cultivar, location, soil nutrients, and the availability of water, may impact the bioactive nutritional value of chestnut fruits. According to research by Poljak et al. [82], small genetic variations and phenotypic adaptations to various climatic circumstances are associated with the morphological and phenological variances among several ecotypes of the Portuguese chestnut.

## 3. Chestnut Adaptative Strategies to Climate Changes

Sort-term adaptation strategies are actions taken by agricultural producers and were described as simple changes in orchard interventions that can be implemented in one or two seasons, such as irrigation management, soil management, cover crops, cultural practices, weather protection, and pest and disease protection [59].

Many parameters must be considered when planning the orchard’s installation, such as soil, climate, density, rootstock, cultivars, pollinators, planting, mulching, cover crops, shelters, and defense against pests. After planting, management should include weed control, pruning, watering, and balanced fertilization [83]. These are examples of strategies that can increase the orchard’s resilience to climate change.

Water stress in plants can lead to a reduction in crop yield, decreased water availability, reduced soil capacities, and a reduction in nutrients provided to the plant. It is important to plan irrigation to avoid crop losses. Through the use of innovative irrigation techniques such as regulated deficit irrigation (RDI) and partial root drying (PRD), among others, currently, agronomists are investigating new ways to maximize the efficiency of crop water consumption [84]. The RDI technique is one way of maximizing water use efficiency (WUE) for higher yields per unit of irrigation water: the crop is exposed to a certain level of water stress either during a particular period or throughout the whole growing season [85]. To use the PRD technique, a customized irrigation system must be set up so that 50% of the root system is always wet and 50% is always dry. Root development is accelerated by PRD in deeper soil layers [59].

Irrigation is an effective way to enhance species hydration and is a good climate change adaptation technique, but it comes with new costs for businesses, which farmers should consider [59]. Only 23% of the 835 ha planted between 2007 and 2013 were irrigated, and in 2018, just 447 ha in Portugal were irrigated. Chestnut orchards younger than 50 years old are frequently irrigated in France.

**Figure 8:**
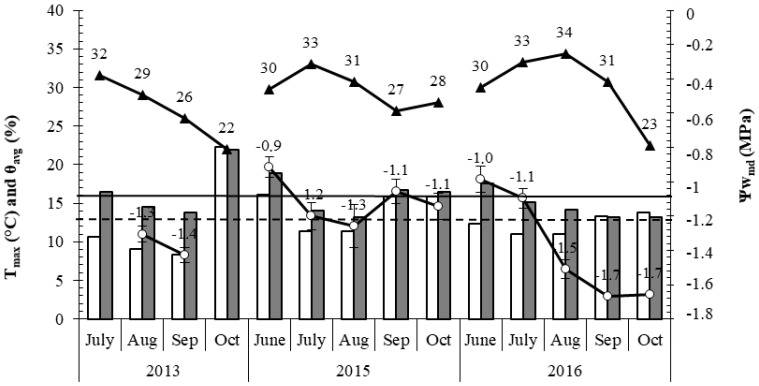
Season pattern of the monthly mean midday stem water potential (Ψw_md_, ○, in MPa); mean of the daily maximum temperature occurred in the measuring days (Tmax, ▲, in °C), and monthly mean soil water content (ϴavg, in %) at the 10 to 40 cm depth (white columns) and at the 50–80 cm depth (grey columns). The dashed line represents the critical branch water potential (below this value will be considered stress for the chestnut tree) determined by the pressure chamber method. Bars are standard error for Fisher test (*p* < 0.05). Adapted from [86].

Mota et al. [44] conducted a trial between 2013 and 2016 to evaluate the impact of irrigation on chestnut cultivation. The study was run in an orchard (planted in 1993) located in Bragança county (which represents 23% of the Portuguese area of chestnut) characterized by cambisoils with 100 cm of thickness and 3% organic matter in a 10 cm–60 cm layer. As shown in Figure 8, it and may represent the typical situation of the orchards in Portugal in the summertime (July to September). The soil water content (θavg) may easily decrease below the wilting point in both layers, 10 cm–40 cm (WP = 13%) and 50 cm–80 cm (WP = 16%). These drought conditions strongly affect tree water relations, inducing a decrease in their level below −1.2 MPa (considered by these authors the threshold level). These drought conditions, coupled with high daily temperatures, adversely affect pollination in July, where temperatures usually exceed 30 °C, and hinder nut growth inside the burr (Figure 8) [84].

According to Mota et al. [44,87] increasing chestnut production per tree by applying irrigation based on tree water potential is enough. The results show that chestnut displayed various patterns of variance in phenotypic expression after growing in two different water regimes, with 50% and 90% of full irrigation, according to [34]. Furthermore, the fertirrigation system (a mix of irrigation and fertilization) can improve plant irrigation, nutrition, and quality. Because chestnut production was 22–37 percent higher in irrigated trees compared to non-irrigated trees, and according to Mota et al. [44], irrigation is a factor that boosts production. Moreover, some research has shown that irrigation affects the size index, fruit weight, and production per tree of chestnut trees. Irrigation boosts the commercial value of chestnuts by increasing their size while maintaining their nutritional value and sensory features (fruit size, firmness, tastiness, and sweetness), while having no negative impact on the chemical makeup of the chestnut [44,88].

The removal of cover crops and organic wastes leads to a reduction in soil organic carbon and an increase in soil erosion, highlighting the detrimental effects of these practices [89]. Michalopoulos et al., instead of the conventional spontaneous vegetation, suggested using seed mix cover crops, which may increase soil coverage by up to 100% and encourage flora diversification [90]. According to Correia and team [91], leguminous cover crops increase the orchards’ sustainability and profitability. Soil fertility is another crucial adaptation strategy for preserving soil properties and crop development [59]. Research on chestnut fertilization and its potential outcomes is currently uncommon. Internal organic inputs and less soil disturbance increased soil organic carbon (SOC) [92,93].

Concerning climate change mitigation in pests and diseases, Australian producers commonly apply organic chemicals to combat *P. cinnamomi*. So, to enhance yields and create soil similar to undisturbed woodland Australian wetlands, the producers add huge volumes of guano, straw, harvesting waste, and others [94,95,96]. Bacteria such as *Pseudomonas* spp., *Bacillus subtilis* var. *niger*, *Flavobacterium* spp., *Chromobacterium violaceum*, and Actinomycetes that prosper on organic matter [94,96,97] are involved in the lysis of *P. cinnamomi* and mycelium [94,98], as well as the interruption of the new cycles, such as the release of zoospores [99]. Unfortunately, because of the rising price of organic matter, it is becoming increasingly rare in Portuguese chestnut orchards.

Likewise, quick adaptation techniques are required due to the negative effects of extreme weather events and high sun radiation on crops. In response to severe temperatures, careful clone selection was used to choose the more efficient types, providing for the minimum level of damage. Furthermore, protective substances have been developed, such as foliar sprays. Silicone (Si) has been investigated in the case of chestnut species to protect them against harsh events. Si is a nutrient that stimulates the plant’s latent tolerance systems by bio-stimulating plant protection under environmental stress [37]. Other researchers speculate that Si plays a role in establishing a physical barrier that can limit the penetration of fungal hyphae. Alternatively, it may trigger the accumulation of substances with antifungal properties, such as flavonoids and diterpenoid phytoalexins, which can break down the cell walls of fungi and bacteria [100,101]. Moreover, according to Xu et al. [102] phytoliths play a role in controlling the opening and closing of stomata, enhancing the thickness of the cuticle layer, and reducing water loss. These effects were associated with lower transpiration rates and stomatal conductance values. Additionally, the application of Si led to plants having increased levels of saturated lipids, and the Si fertilization-treated plants show a decrease in unsaturated lipids which improved the stability and integrity of their membranes when exposed to high temperatures. In addition, due to an increase in the rigidity of the xylem vessel due to Si treatment improving tolerance to drought and heat, Si having a significant structural and protective role in the plant’s protection is accomplished with minimal energy costs [103]. Plants treated with Si had better recovery capacity when resubmitted to the new period of ideal temperature (25 °C) following the warm phase (32 °C) [104]. Si was also applied to protect the chestnut tree’s leaves, roots, and stems from pathogenic fungus. The influence of Si on chestnut plants infected with *C. parasitica* was discovered, and Si fertilization can reduce the severity of the disease and the mortality rate of chestnut plants. The study indicated that the effect of Si in chestnut trees infected with *P. cinnamomi* had some changes in phenolic extracts from plants treated with various Si concentrations and exhibited antifungal action when exposed to the oomycete [48]. Si was also applied to protect the chestnut tree’s leaves, roots, and stems from pathogenic fungus. The influence of Si on chestnut plants infected with chestnut blight (*C. parasitica*) was also discovered to reduce the severity of the disease and the mortality rate of chestnut plants. According to numerous research studies, Si is beneficial for plant survival when faced with disease, and it can also increase plant resistance to diseases [101,105].

In the past century, numerous initiatives were launched that were aimed at comprehending the intricate relationships involving the cellular, molecular, and genetic aspects underlying both the biotic and abiotic interactions related to chestnuts [27]. Breeding methods should concentrate on choosing clones with high resistance to heat and water stress. Some cultivars of chestnut have been shown to be more resistant to hot weather and/or water stress. Cultivar and clonal selection may also consider resistance to pests and diseases, minimizing the overuse of pesticides and herbicides that could endanger the safety of drinking water [42].

Tolerance to diseases and pests may be an important factor in the cultivar and rootstock selection to be installed in new chestnut orchards, as, among other reasons, they allow for less pesticide and herbicide use in the fight against pests and diseases [59]. For instance, the knowledge and understanding of the basic genetic structure of ink disease resistance in the chestnut tree can improve the precision of disease resistance genomic selection [106].

The production of resistant hybrid rootstocks, as a result of controlled pollination between the species *C. sativa* and the resistant species *C. crenata* and *C. mollissima*, has become extremely important as the outcome [27]. The Euro-Japanese hybrids chosen in France by the French National Institute for Agronomic Research, ‘Institut National de la Recherche Agronomique’ (INRA), in the second half of the 20th century is still the most often used clonal rootstocks. These hybrids can be quickly multiplied through layering or soft cuttings. The INRA hybrid rootstocks are genetically compatible with most *C. sativa* cultivars and tolerant to *Phytophthora* spp. (causes ink disease), but moderately sensitive to *C. parasitica* (causes blight disease). Popular rootstocks include Ca-07 ‘Marsol’ (moderate *Phytophthora* spp. resistance), Ca-74 ‘Maraval’ (*Phytophthora* spp. resistance, low vigor and moderate drought tolerance), Ca-118 ‘Marlhac’ (moderate *Phytophthora* spp. resistance, able to grow at temperatures below 10 °C), and Ca-90 ‘Ferosacre’ (*Phytophthora* spp. resistance, but sensitive to temperatures below 10 °C) [67].

Similar to other European countries, Portugal began a breeding effort in the middle of the 20th century under the direction of Columbano Taveira Fernandes to create hybrid plants. *C. sativa* and the disease-resistant Chinese and Japanese species were hybridized with the aim to obtain new genotypes with ink disease resistance [107]. Some of these clones were transported to the University of Trás-os-Montes and Alto Douro (UTAD) in the early 1980s; one of them was chosen and given the name ColUTAD^®^. ColUTAD^®^ is a new clonal rootstock developed through the crossbreeding of *C. crenata* and *C. sativa* that was chosen and born in Portugal to better withstand the climatic conditions and, in particular, to increase the photosynthetic efficiency at high temperatures. ColUTAD^®^ is a good choice for planting in places where there may be water stagnation since it has a high resistance to ink disease and medium resistance to early frosts. As is usual with small rootstocks, this rootstock needs rich, deep soils [19]. It also presents a good compatibility for grafting with *C. sativa* cultivars or can be crop as a direct producer. The vegetative propagated methods are conventional propagation and micropropagation, with micropropagation being more efficient in large-scale production of individual genotypes [108].

More recently, since the beginning of the 21st century, the research team coordinated by Rita Costa in collaboration with University of Trás-os-Montes and Alto Douro [109] developed a new breeding program with the aim to obtain new genotypes better adapted not only to ink disease, but also to drought and heat stresses.

Over the years, chestnut farmers have chosen the cultivars that are most suited to each location’s climate. However, growers would likely need to switch out sensitive cultivars for more climate-resilient ones because of future climate change. Climate change may dramatically influence how chestnut tree cultivars are distributed. In this sense, a project was developed in Portugal: ClimCast, “The new challenges for the orchard in the context of climate change” whose main objectives were: (i) to characterize the evolution of edaphoclimatic conditions in the main producing regions and other regions in terms of potential for chestnut production; (ii) identify the varieties best adapted to future climate conditions; (iii) develop tools to estimate future production; (iv) develop a manual of good chestnut cultivation practices to be adopted by producers; and (v) create a chestnut warning network. Thus, the results of this study currently make it possible to alert producers to the adaptive specificity of varieties, offering indications on the most suitable varieties for different climatic situations and also suggesting the introduction of varieties from some regions into others. Furthermore, cultural models better suited to contrasting soil and climate situations were proposed to producers [110].

Comparative information was also made available for all the varieties studied about the timing of their phenological states and their respective growing degree day (°D) requirements. This parameter will help in the temporal prediction of different phenological states and will be an extremely important instrument to help adjust the choice of cultivar per location. Monitoring soil evolution makes it possible to assess the impact of climate change on the soil and make the necessary adjustments to fertilization plans. A climate productivity model and potential chestnut production chart will be developed in Portugal. This model will make it possible to better systematize knowledge about the climatic conditions to better production and, in advance, help to predict the annual production of chestnuts, an aspect of the greatest importance for the industry [111].

Actual yield variation represents the interaction of climatic conditions, management, technology, and elevated atmospheric CO_2_ concentrations [112]. Increasing net photosynthesis and reducing stomatal conductance in plants exposed to increasing CO_2_ can have a positive impact on their development and physiology, which increases radiation and water usage efficiency [113]. However, depending on the type of plant, increased CO_2_ concentrations may also encourage weed growth, increasing the competition for water and soil nutrients. Therefore, understanding how CO_2_ influences primary plant output is crucial for assessing the effects of climate change [114].

Now, modern crop breeding strategies incorporate the integration of omics and other genomics technologies. While emerging technologies such as genome editing may help with the controlled production of better cultivars, their practical application is still constrained by concerns with public perception and regulation [115].

Given the current climate change prediction models in Europe, researchers and chestnut producers are increasingly discussing and expressing concerns about the translocations and reintroductions of chestnut cultivars. It is not surprising that cultivars that currently grow in Southern Europe appear in Central and Northern Europe [15,116].

## 4. Conclusions

There is a substantial concern regarding food security and economic losses caused by extreme weather occurrences, as has already been underlined by other authors.

Climate change adversely affected chestnut trees through altered phenology, and physiology, reduced biodiversity, and genetic characteristics changed. Although the biodiversity and acclimatization characteristics of the chestnut tree are vital for their survival, they may not be sufficient to face the new challenges imposed by climate change. As we observed in the article, some cultivars, such as Judia, despite possessing, for instance, thick palisade parenchyma, were found to benefit from this adaptation only in optimal plant conditions. In less suitable locations, however, this cultivar was the one that had the most difficulties surviving during periods of drought and heat. However, Longal is a cultivar that seems to be more resilient because it was demonstrated to withstand more demanding conditions more successfully, even if it does not have the same noticeable parenchyma thickness as Judia. Future climate projections based on credible socioeconomic scenarios provide valuable insights for developing effective adaptation strategies.

Fluctuations in temperature and shifts in rainfall patterns serve as critical indicators of environmental stress for chestnut trees. Implementing strategies such as irrigation and protective compounds, which have been studied for their effectiveness in mitigating climate change impacts, is essential for both short-term and long-term adaptation. Utilizing breeding techniques is crucial for cultivating climate-resilient crops that can withstand challenges according to heat and drought conditions.

Therefore, scientists must prioritize maximizing plant growth and development in the face of abiotic challenges. Resistance to biotic and abiotic problems, the spread of innovative biological techniques, the implementation of diverse cropping patterns, and various conventional and non-conventional ways will be employed in the future to rescue agriculture and maintain a stable chestnut sector. In order to address climate change, it will be essential to develop environmentally friendly crops through genome editing using the CRISPR system.

Future studies should address mitigation strategies and governance programs, taking specific attention to the developing world. These results should motivate researchers to pursue understudied areas, advancing the field of science.

## Figures and Tables

**Table 1:** Average chlorophyll and carotenoid composition in leaves harvested from the south side of the canopy of 15-year-old trees in the germplasm bank in Vila Real, Portugal. Adapted from [66].

Cultivar	Chlorophyll (μg·cm^−2^)	Carotenoids (μg·cm^−2^)	Total (μg·cm^−2^)	Cla/Clb	Cl/Car
Judia	113.3	29.3	142.6	3.60	3.87
Longal	103.0	26.3	129.3	4.01	3.92
Martaínha	85.7	19.5	105.2	4.35	4.39

**Table 2:** Influence of irrigation mineral macronutrient and micronutrient content on leaves from irrigated trees (TI) and non-irrigated trees (NI) of Judia cultivar located in an orchard in Bragança, Portugal. Leaves were collected monthly between July and October. Mean soil water at 10–40 cm deep was 12.9% and 11.8% (NI), and 17.1% and 16.4% (TI), respectively. Adapted from [46].

	Treatments	Macronutrients (g kg^−1^)						Micronutrients (mg kg^−1^)				
		N	P	K	Ca	Mg	S	B	Fe	Zn	Mn	Cu
Average	NI	19.5	2.5	11.5	7.2	2.1	0.7	29.0	25.0	19.8	718.0	10.8
	TI	21.5	2.6	12.8	7.2	2.2	0.7	29.3	21.0	26.0	640.8	9.80
Variation (%)	NI	100.0	100.0	100.0	100.0	100.0	100.0	100.0	100.0	100.0	100.0	100.0
	TI	109.9	106.1	110.9	100.0	106.0	100.0	100.9	84.00	131.6	89.20	90.70
